# Supplementary material for: Stimulus type shapes the topology of cellular functional networks in mouse visual cortex
Source: Nat Commun. 2024 Jul 9;15:5753. doi: 10.1038/s41467-024-49704-0 (PMC11233648; doi:10.1038/s41467-024-49704-0)
Supplement: Supplementary file 1 — Supplementary Information [file 41467_2024_49704_MOESM1_ESM.pdf]

# Supplementary Information

## Stimulus type shapes the topology of cellular functional networks in mouse visual cortex

**Disheng Tang[1,2,3 *], Joel Zylberberg[4,5 *†], Xiaoxuan Jia[1,3,6 *†], Hannah Choi[2,7 *†]**

[1]School of Life Sciences, Tsinghua University, Beijing, 100084, PR China
[2]Quantitative Biosciences Program, Georgia Institute of Technology, Atlanta, 30332, GA, USA
[3]IDG/McGovern Institute for Brain Research, Tsinghua University, Beijing, 100084, PR China
[4]Department of Physics and Astronomy, and Centre for Vision Research, York University, Toronto, ON M3J 1P3, Ontario, Canada
[5]Learning in Machines and Brains Program, CIFAR, Toronto, ON M5G 1M1, Ontario, Canada
[6] Tsinghua–Peking Center for Life Sciences, Tsinghua University, Beijing, 100084, PR China
[7]School of Mathematics, Georgia Institute of Technology, Atlanta, 30332, GA, USA

*Corresponding authors. E-mails: dishengtang3@gmail.com; joelzy@yorku.ca; jxiaoxuan@gmail.com; hannahch@gatech.edu
†These authors contributed equally to this work.

**This PDF file includes**

Figs. S1 to S17

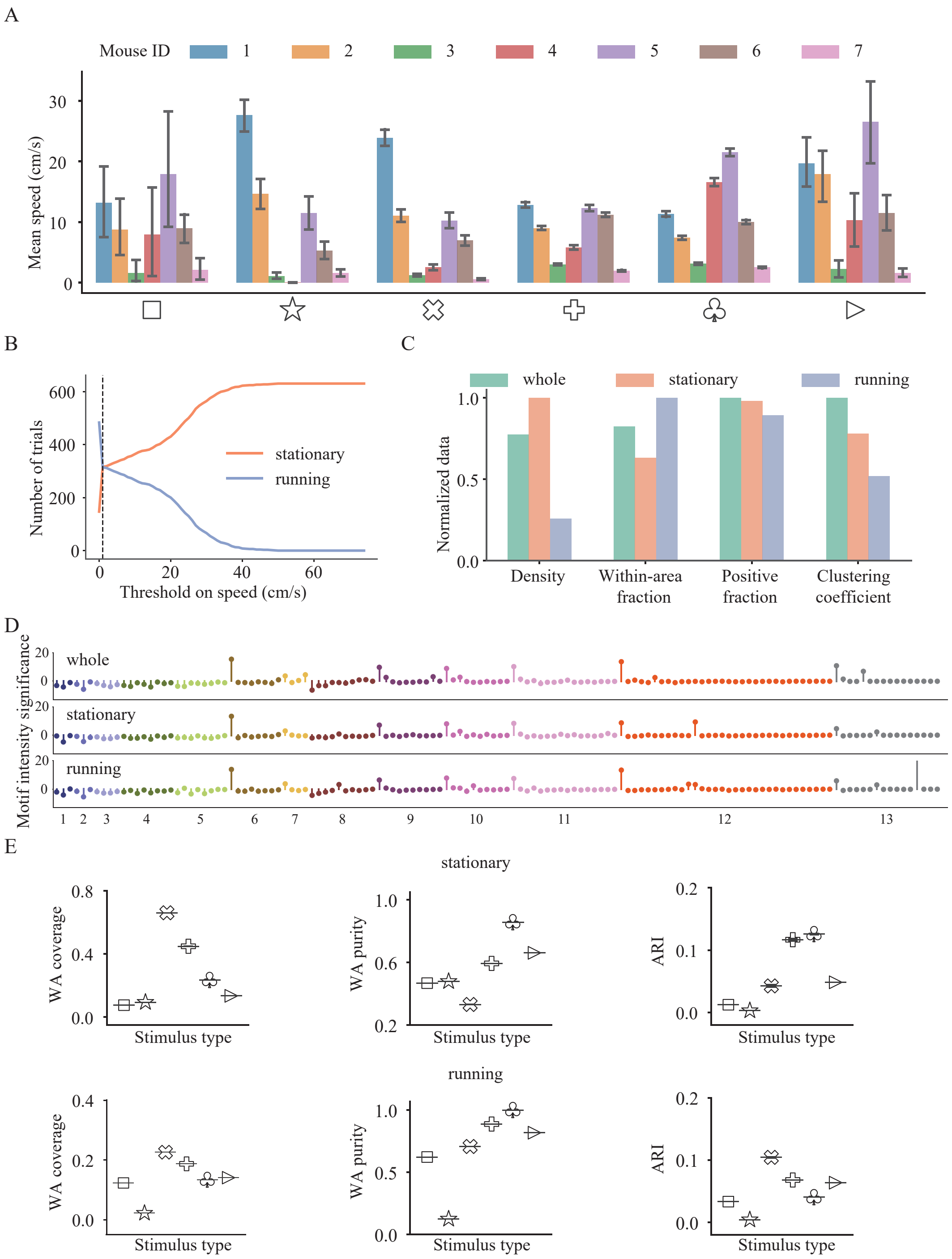


**Fig.** S1 | Influence of running speed on functional networks. (A) Distribution of mean speed for each mouse (session) during all visual stimuli. Error bars show 95% confidence interval across the mean speed of all trials. Each color corresponds to an individual mouse. (B) Number of stationary and running trials against the threshold on speed. Data from session 2 during drifting gratings are selected for subsequent analyses due to the comparable number of trials for stationary and running periods. The optimal threshold (1 cm/s) is selected to differentiate stationary and running phases based on the maximal change induced in their number of trials, consistent with the threshold used in previous work [1]. (C) Basic network properties of functional networks of all, stationary and running trials for session 2 during drifting gratings. All metrics are normalized by the maximum for better comparison. (D) Motif significance sequences for networks of all, stationary and running trials for session 2 during drifting gratings as a representative example. (E) WA coverage, WA purity and ARI of stationary and running trials for different stimulus types during session 2. Error bars represent 95% confidence interval, $n = 200$ independent runs of modified Louvain method.

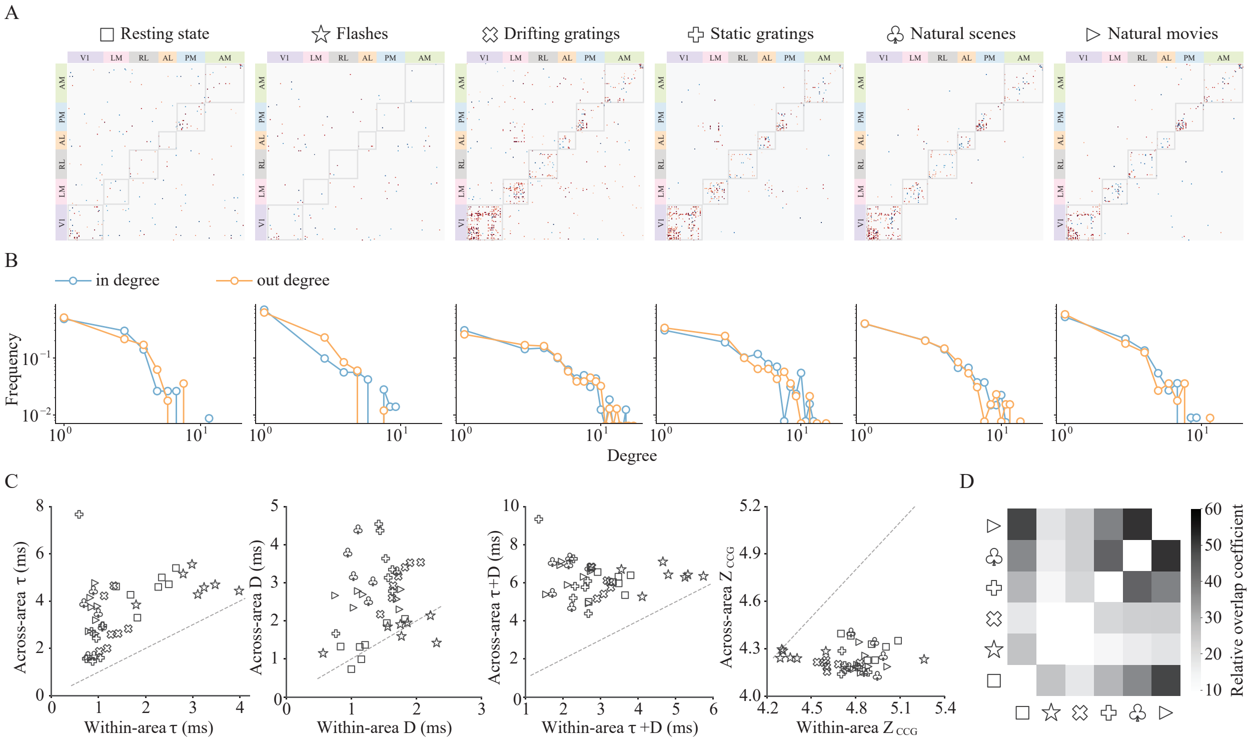


**Fig.** S2 | Basic properties of functional networks during all visual stimuli. (A) CCG adjacency matrices of a mouse given distinct stimuli (on the same scale as in Fig. 1D). (B) Directed degree distributions of a mouse given all stimuli. (C) Across-area *vs* within-area comparisons for the lag $\tau$, duration $D$ and their sum $\tau + D$. (D) Relative overlap coefficient for functional connections during different visual stimulus types. To remove density dependence, we used relative overlap coefficient, defined as overlap coefficient divided by expectation of two random networks with identical densities, to measure the similarity between connections.

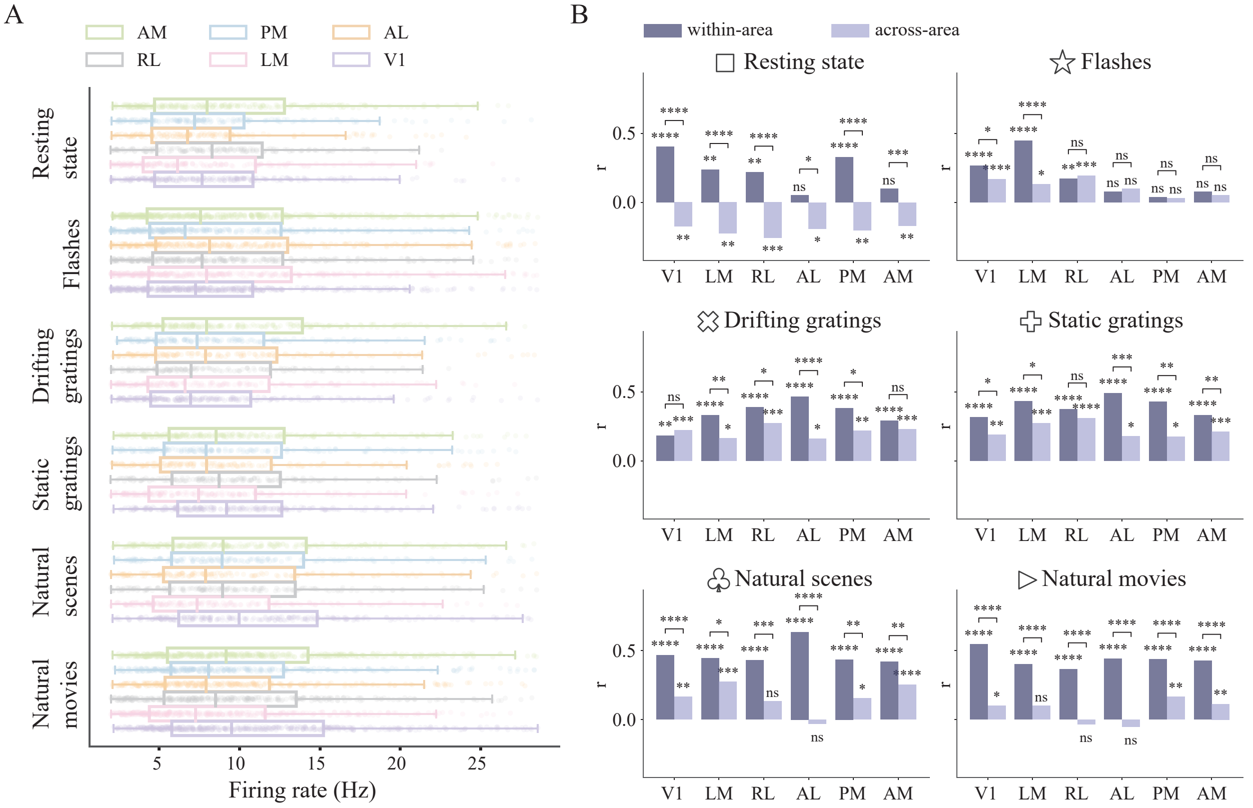


**Fig.** S3 | Firing rate and its relationship with functional connection. (A) Firing rate distributions for each visual area during all visual stimuli. (B) Correlation coefficient between a neuron's firing rate and its number of within-area/across-area functional connections. ns $p > 0.05$, $*p < 0.05$, $**p < 0.01$, $***p < 0.001$, $****p < 0.0001$, Wald test for each correlation value, modified asymptotic (MA) test [2] for comparing overlapping correlations (firing rate as the common variable), both tests are two-sided.

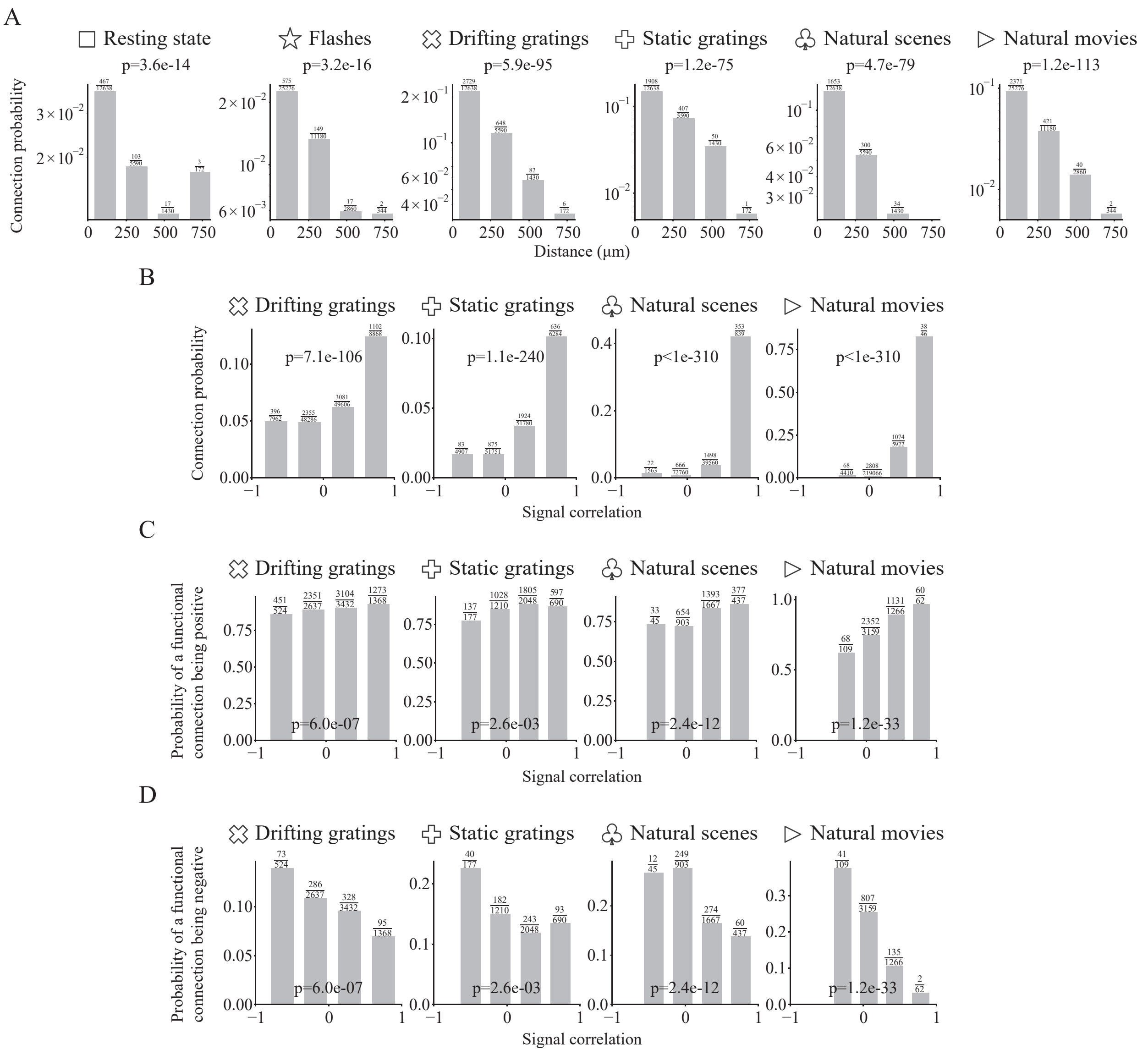


**Fig.** S4 | Two-sided Cochran-Armitage trend test for association between (A) (functional) connection probability and distance of neurons. The rest of the plots show the two-sided Cochran-Armitage trend test for association between signal correlation and (B) (functional) connection probability, (C) probability of a functional connection being positive and (D) probability of a functional connection being negative, under four different types of visual stimuli. Here we excluded flashes since there are only two stimulus conditions (light or dark) and signal correlation could be heavily biased and trivial.

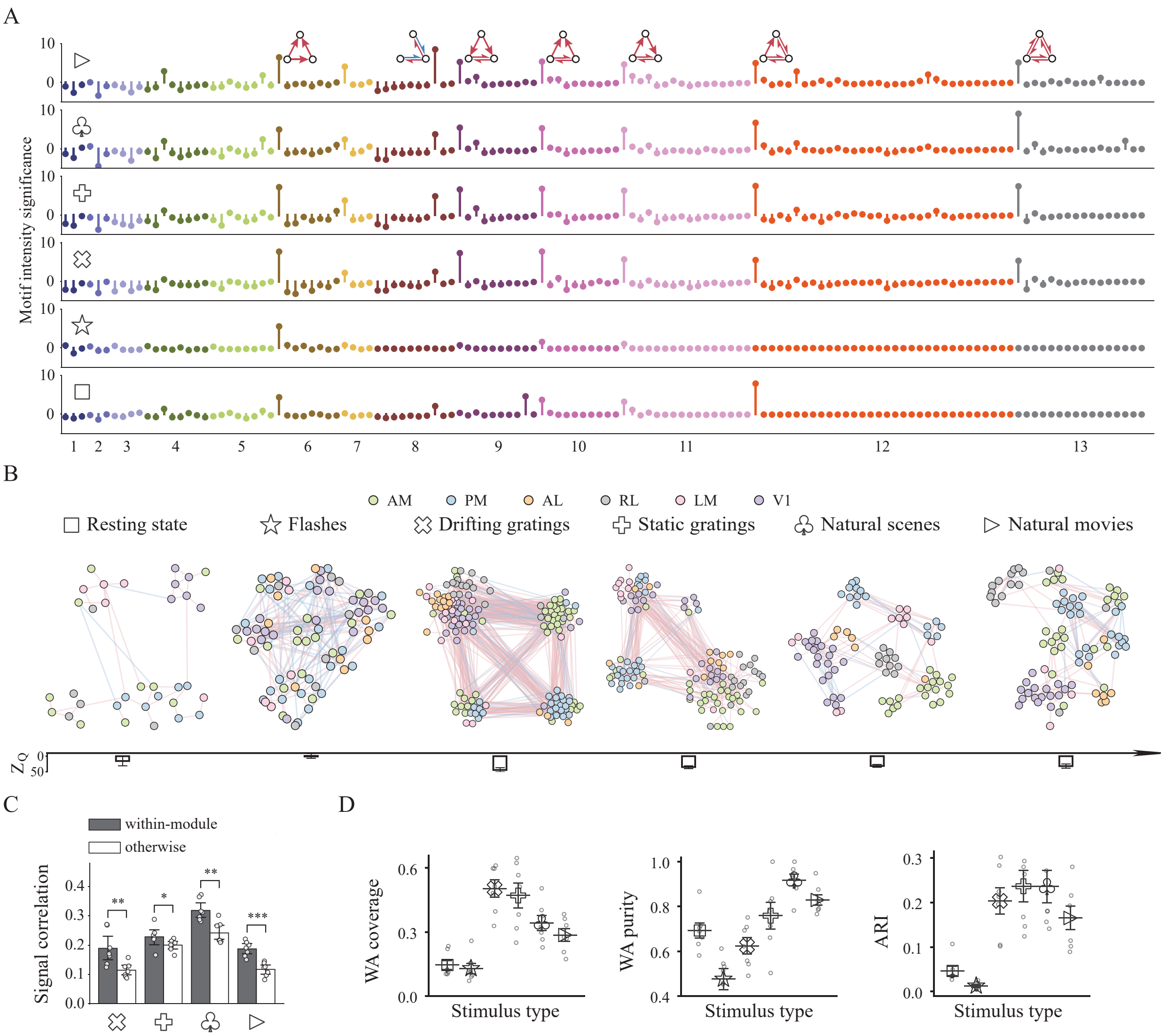


**Fig.** S5 | Network properties using anatomical-distance-preserving model. Only neurons with recorded anatomical locations are included. (A) Motif sequence of the networks during 6 visual stimuli using anatomical-distance-preserving model. (B) Topological structure of functional connectivity of a mouse during six types of visual stimuli with neurons colored by area. (C) Signal correlation for within-module and otherwise connections. $*p < 0.05$, $**p < 0.01$, $***p < 0.001$, rank-sum test, one-sided, $n = 7$ mice. Error bars represent 95% confidence interval. (D) WA coverage, WA purity and ARI during six visual stimuli. Error bars represent standard error of the mean and are displayed with mean, $n = 7$ mice.

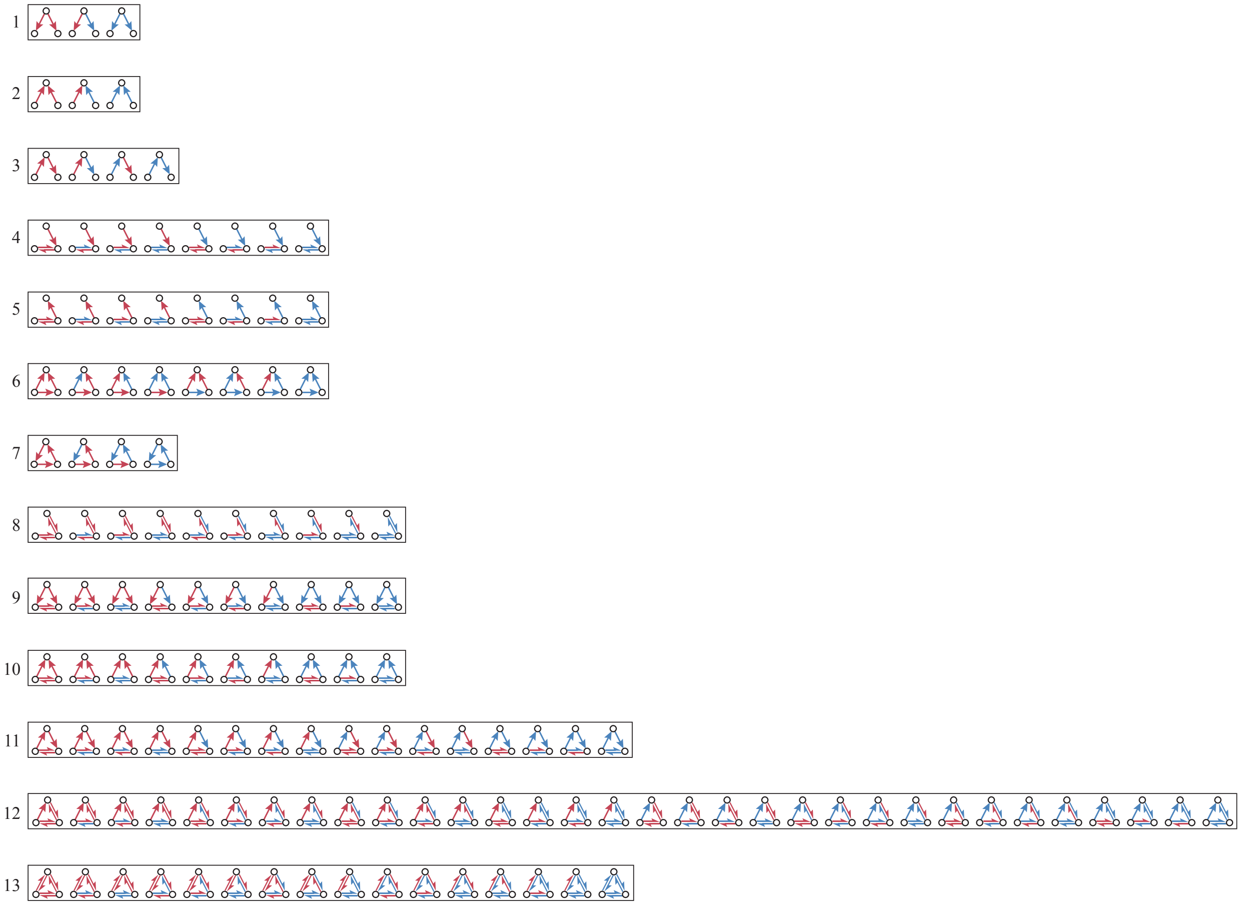


**Fig.** S6 | All 132 signed motifs in the same order as Fig. 2D.

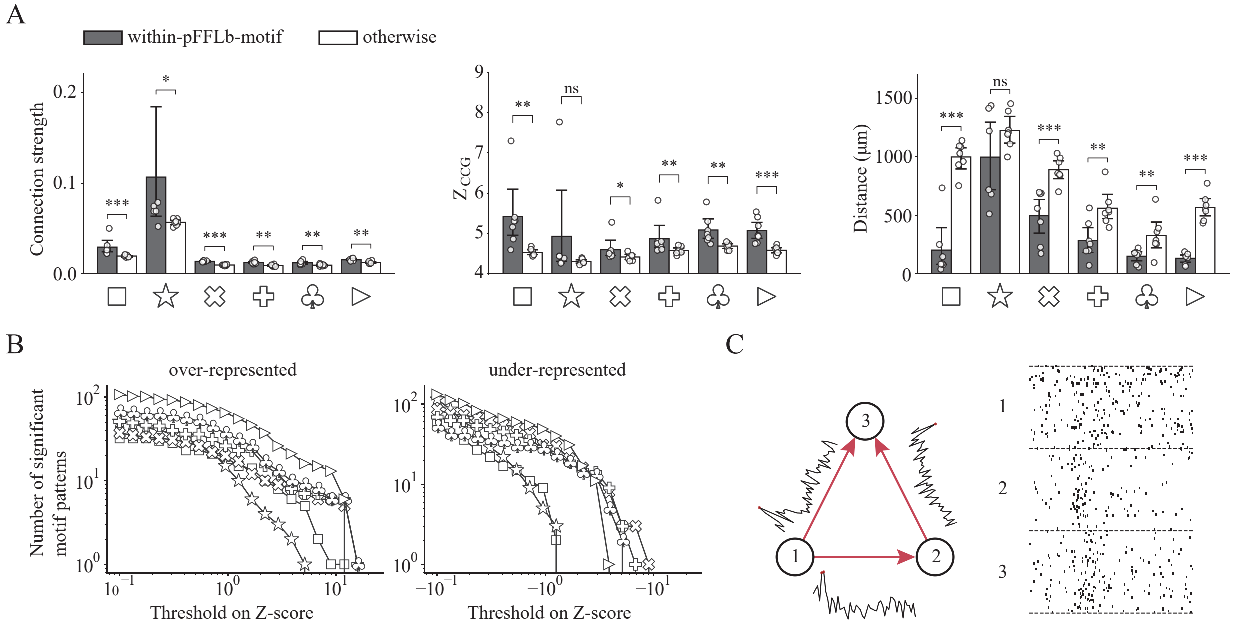


**Fig.** S7 | Further comparison of within-pFFLb-motif and other connections. (A) (left) Connection strength, (center) Z-score of CCG and (right) physical distance for within-pFFLb-motif connections and others during all visual stimuli. ns $p > 0.05$, $*p < 0.05$, $**p < 0.01$, $***p < 0.001$, rank-sum test, one-sided, $n = 7$ mice. Error bars represent 95% confidence interval. (B) Number of significant signed motifs with intensity Z-score higher than the threshold (over-represented) or lower than the threshold (under-represented) during all visual stimuli. (C) Example pFFL motif with CCGs for each connection and spike trains for each neuron.

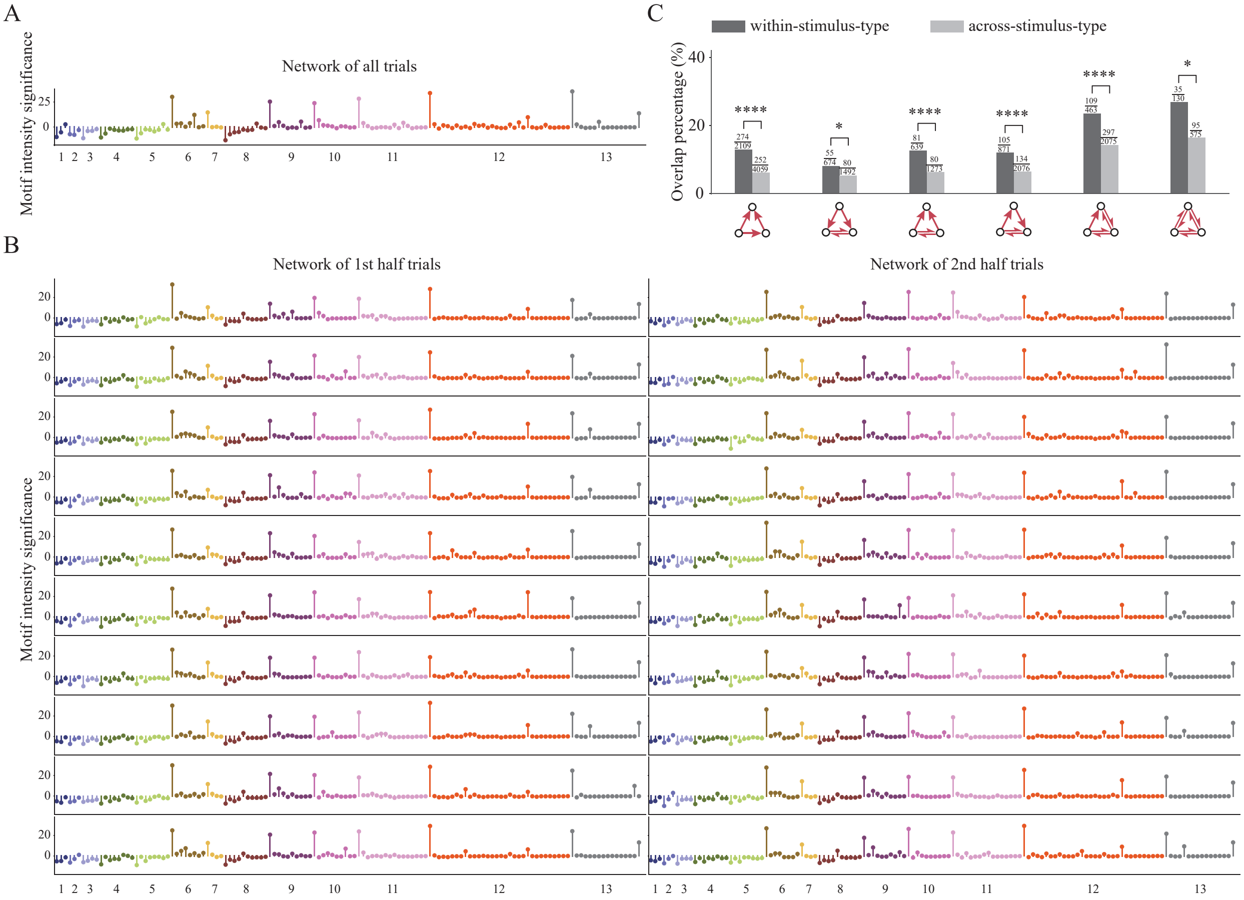


**Fig.** S8 | Functional networks constructed from different trials for example session 7. (A) Motif sequence of the network constructed from all trials of natural scenes. (B) Motif sequences of the networks constructed from two halves of the trials of natural scenes. Each row corresponds to a realization of the random split. For each random split, two halves contain identical repeats of each image to control for stimulus-induced variances. (C) Overlap percentage for pFFLb motifs. For each pFFLb motif, overlap percentage is defined as the percentage of unique motifs that are observed in at least two networks. Overlap percentage has only two categories (observed in more than one network/only one network). The number of samples for within- and across-stimulus-type networks is matched for fair comparison. $*p < 0.05$, $****p < 0.0001$, Chi-squared test, adjusted using Benjamini/Hochberg method.

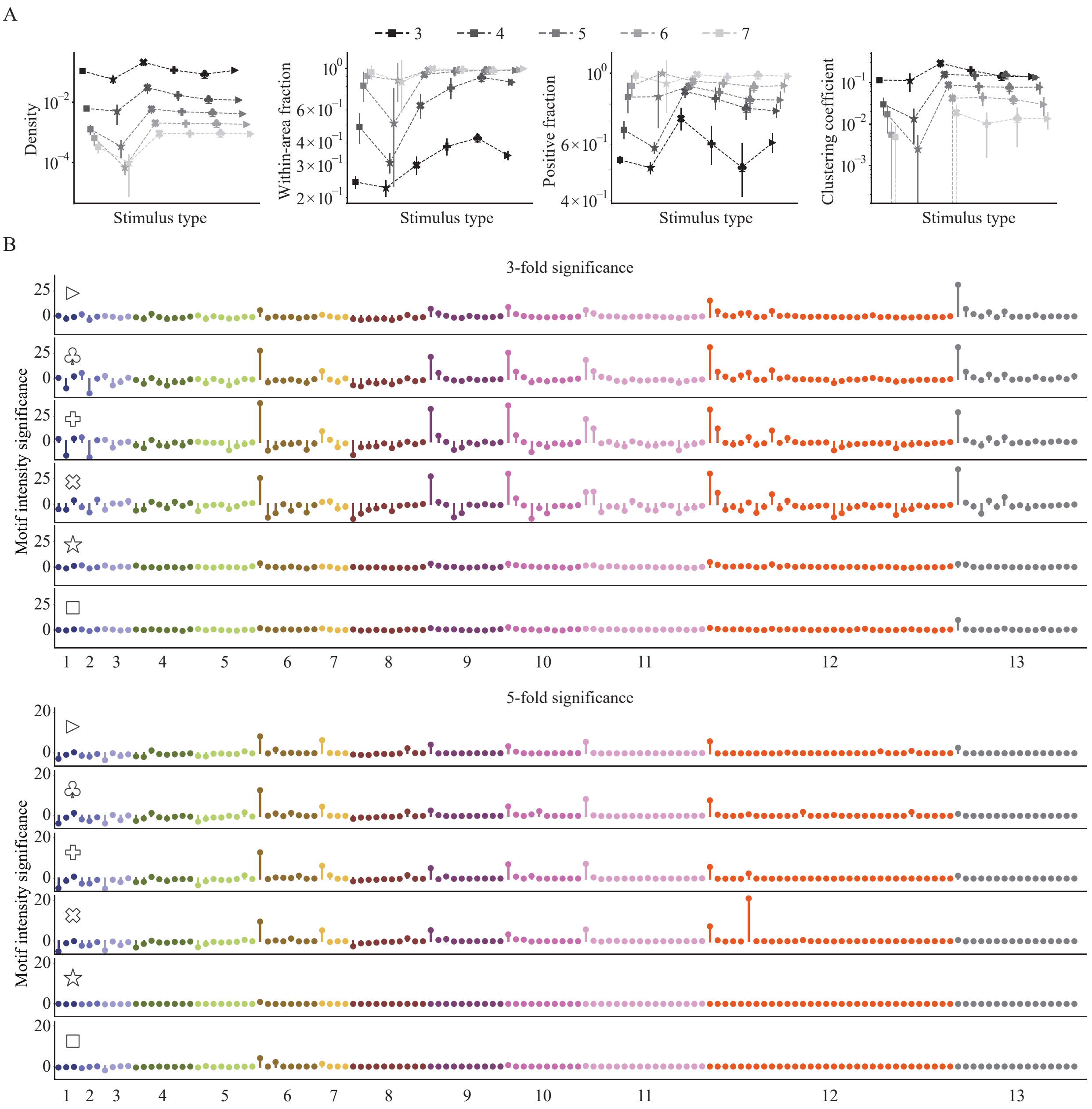


**Fig.** S9 | Functional networks constructed using different significance levels on functional connections. (A) Fundamental properties of the network on different significance levels (from 3-fold to 7-fold). (B) Motif sequences of the networks on different significance levels. Only 3-fold and 5-fold are shown since other significance levels lead to extremely dense or sparse networks. See Fig. 2D for results on 4-fold significance.

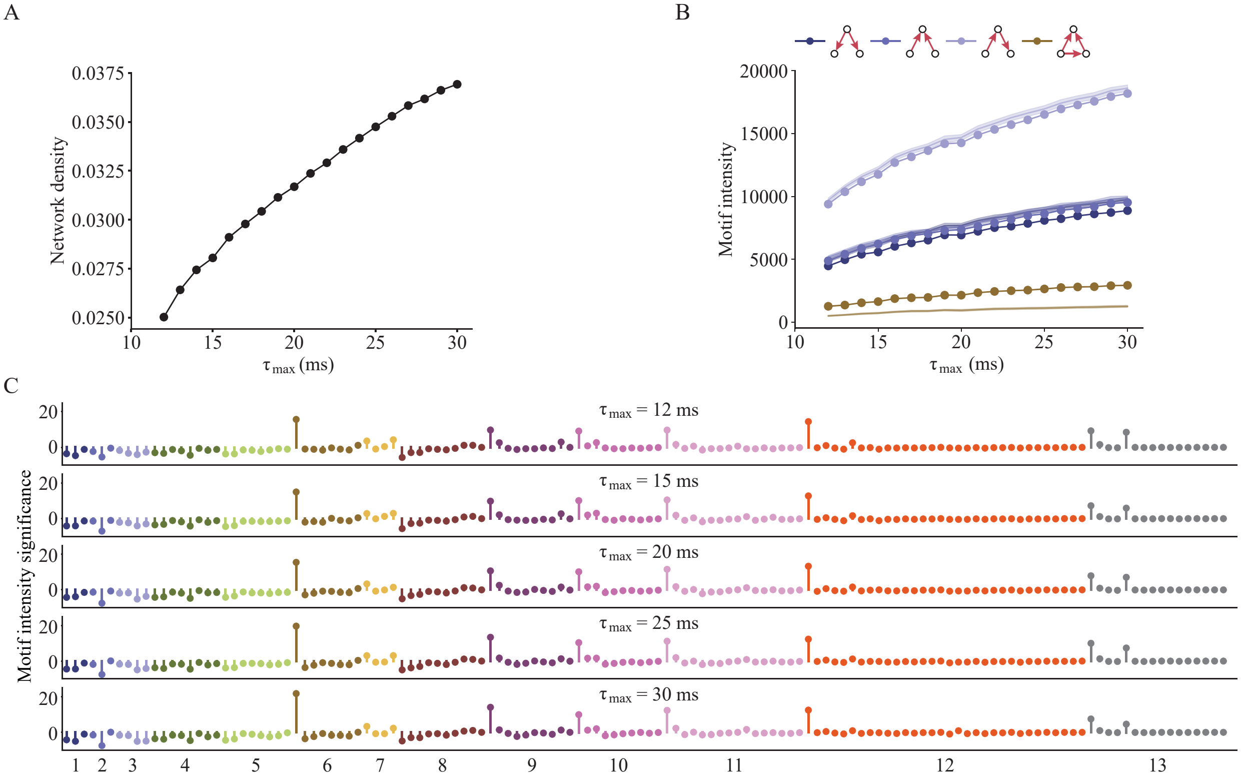


**Fig.** S10 | Impact of CCG window size. Session 2 recordings during drifting grating presentations are illustrated as representative examples in this figure. (A) Network density against the window size of CCG ($\tau_{max}$). (B) Motif intensity for motif ID=1, 2, 3 and 6 against window size of CCG ($\tau_{max}$). Note that motif ID=1, 2, and 3 can be considered as unclosed counterparts of motif ID=6. Shaded areas with mean denoted represent the standard deviation of intensity distribution of the corresponding motifs in surrogate networks generated using Signed-pair-preserving model. (C) Motif intensity significance sequences with different window sizes of CCG.

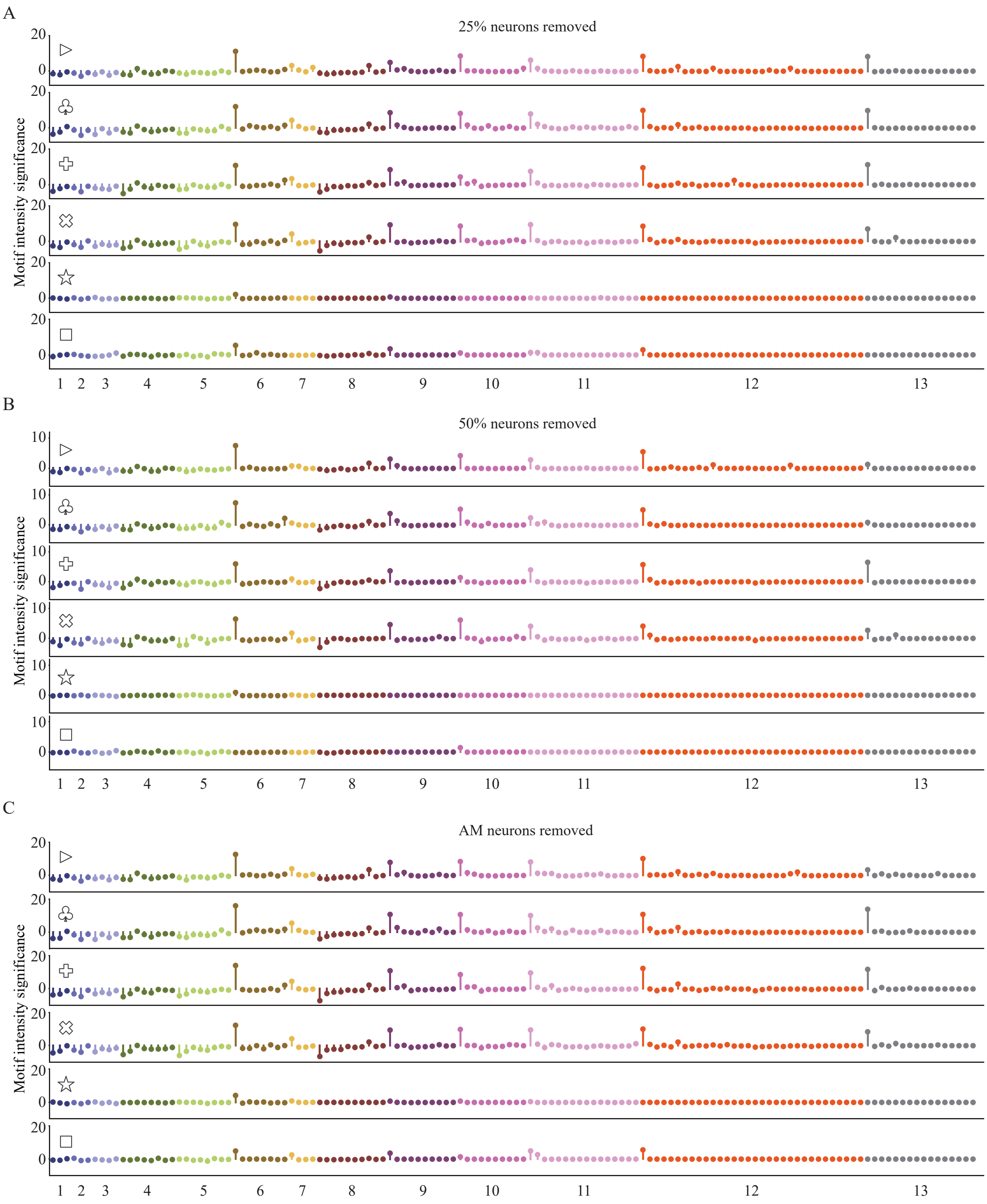


**Fig.** S11 | Motif intensity significance sequences of all signed motifs for subnetworks that are generated by randomly removing (A) 25% of neurons, (B) 50% of neurons, (C) all neurons from AM to simulate the joint-modulation from unobserved units.

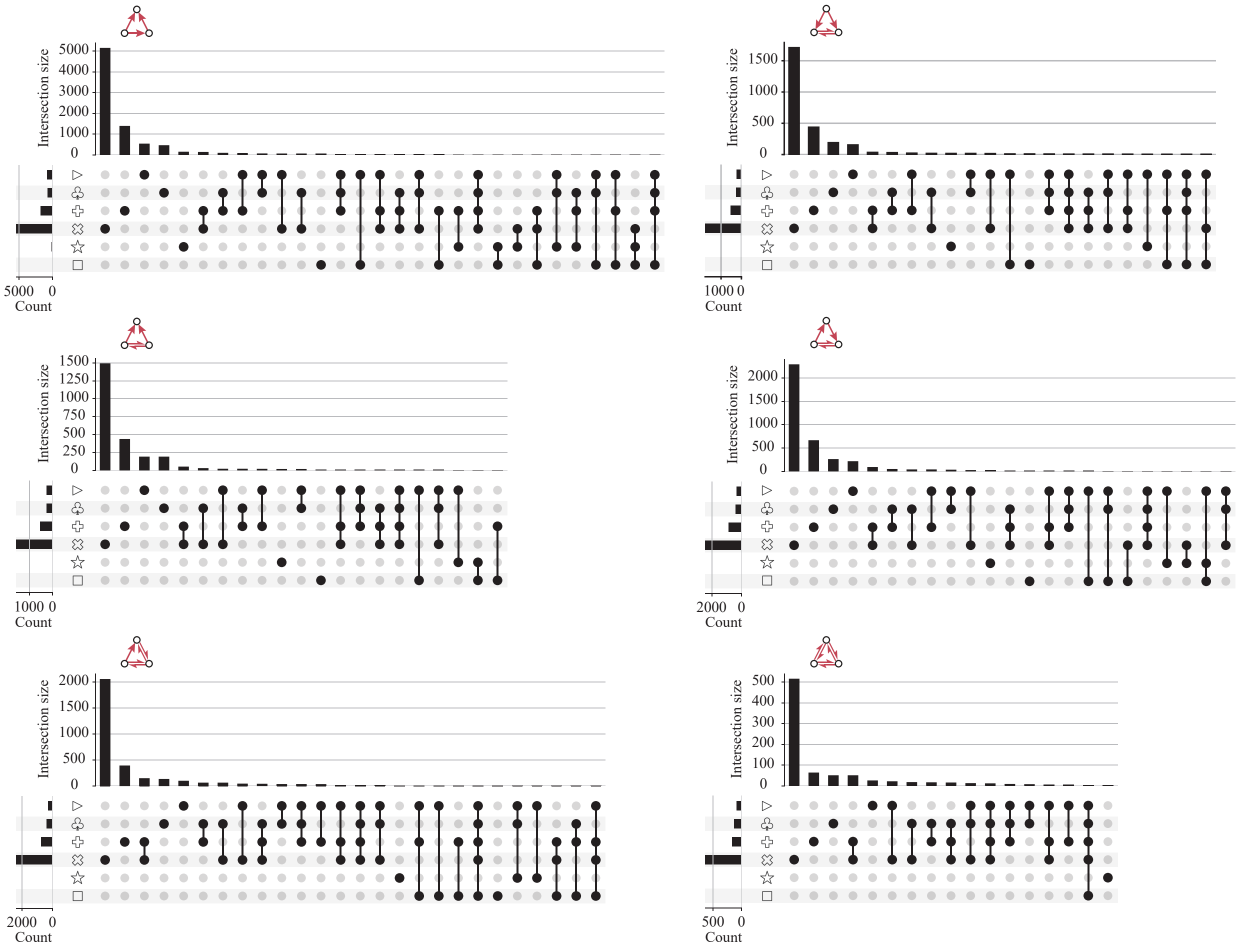


**Fig.** S12 | Intersections of unique motif sets for pFFLb motifs during six types of stimuli. All intersections with at least 1 element are shown.

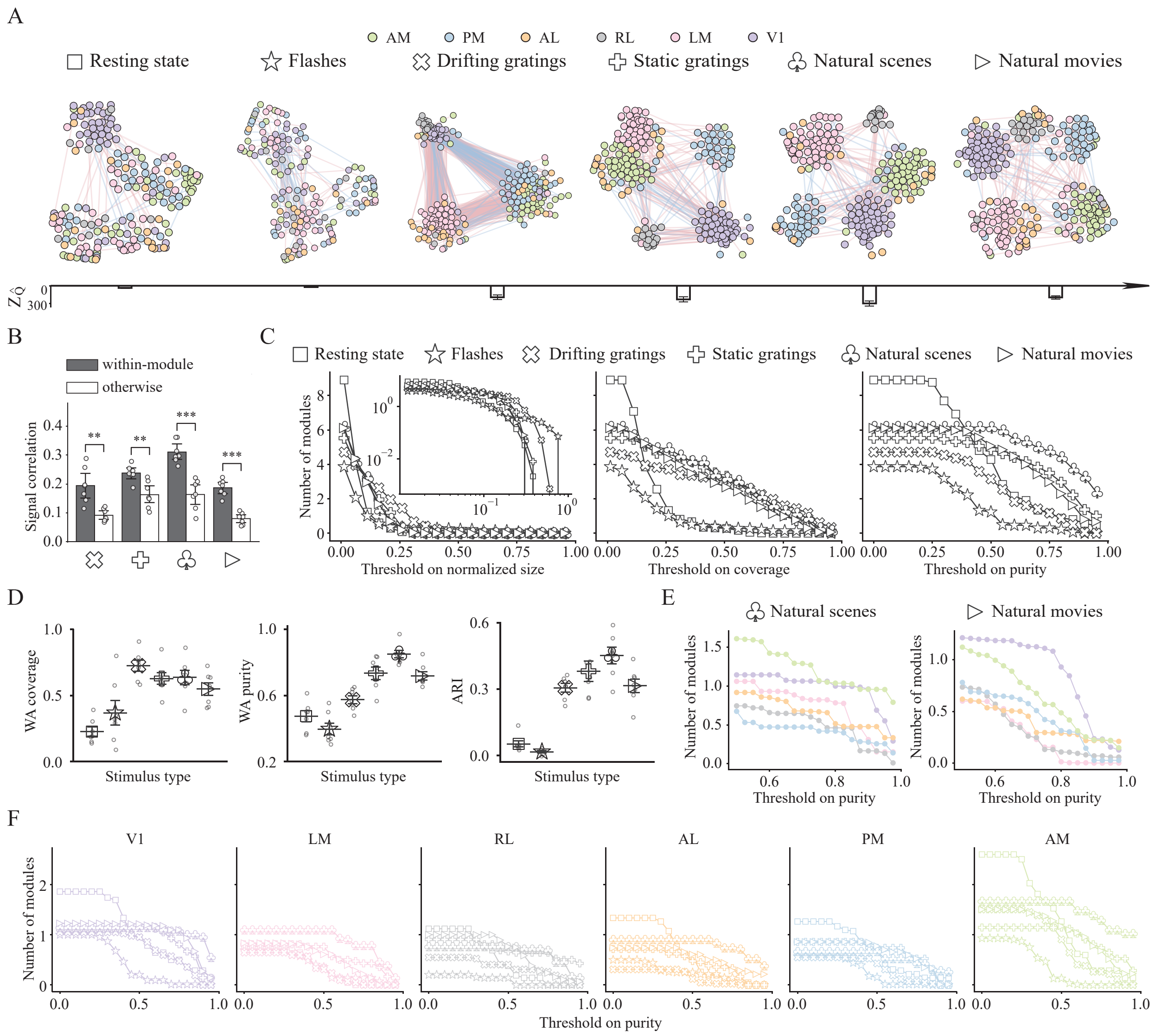


**Fig.** S13 | Results on modular structure with original Modularity by omitting edge signs. (A) Topological structure of functional networks during all visual stimuli. (B) Signal correlation for within-module and other connections. $**p < 10^{-2}$, $***p < 10^{-3}$, rank-sum test, one-sided. (C) Number of modules with normalized size, coverage or purity higher than the threshold, inset shows the plot on a log-log scale. (D) WA (weighted average) coverage, WA purity and ARI during six visual stimuli, the error bars show the 95% confidence intervals, $n = 7$ mice. (E) Number of modules against threshold on purity for each visual area separately during natural scenes and natural movies. (F) Number of modules with purity higher than the threshold for each visual area during all visual stimuli.

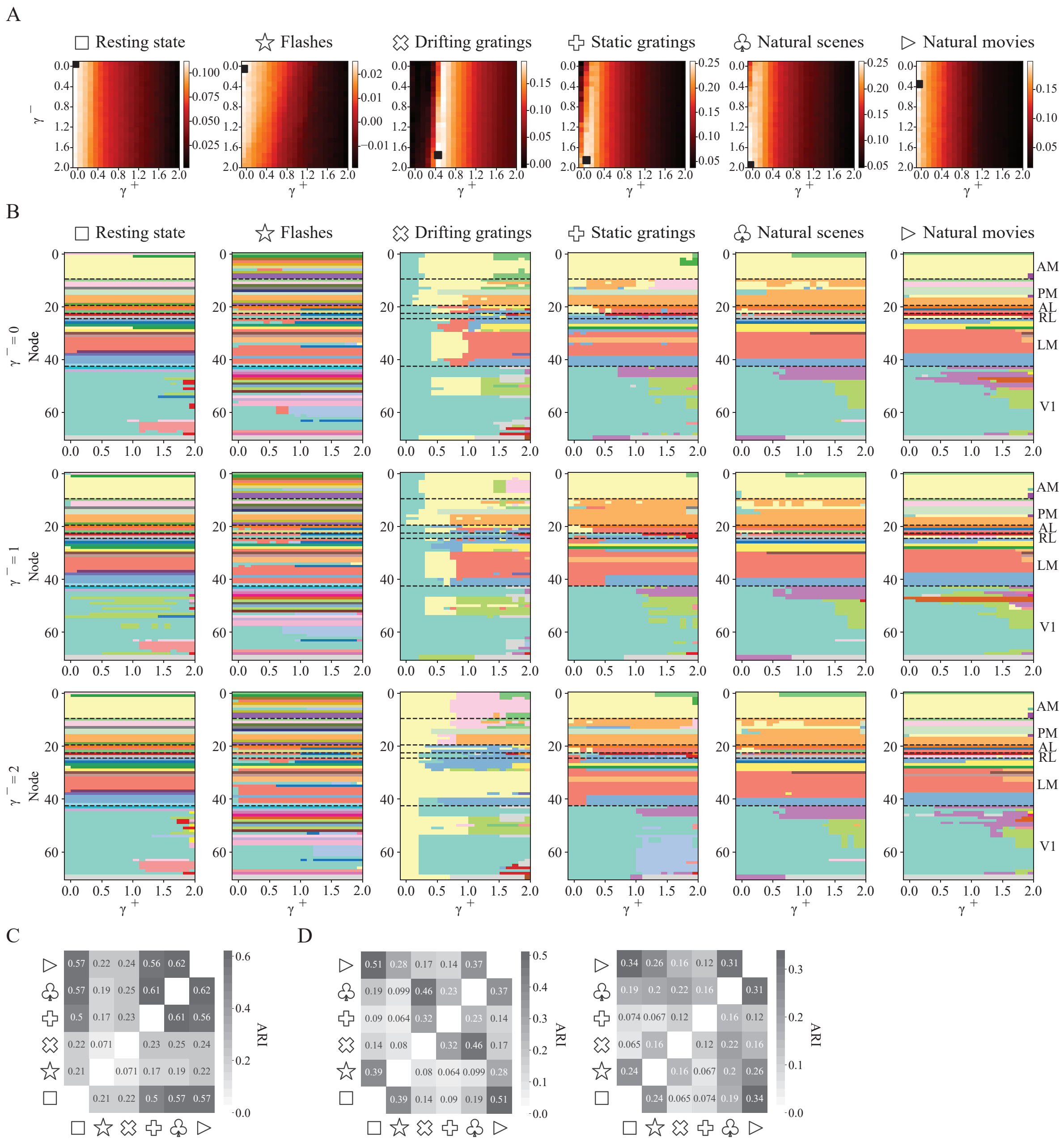

**Fig.** S14 | Multi-resolution modular structure. (A) The Modularity difference heatmap between the empirical network and reference model (Signed-pair-preserving model) is used to determine the resolution parameters $\gamma^+$ and $\gamma^-$, black box represents the maximum of the heatmap while its coordinates correspond to the optimal resolution parameters. Session 7 is used as the illustrative example. (B) Modular partition at different resolution parameters for session 7. Since empirical functional networks tend to have more positive than negative connections, $\gamma^+$ has a larger impact than $\gamma^-$ thus we only show the results obtained with three different values of $\gamma^-$. Module IDs (colors) across different $\gamma^+$ are determined by assigning each module the ID of its largest submodule at the previous step (larger resolution parameter), similar to the previous method [3]. Only neurons with at least one positive connection during any stimuli are included for brevity, and remaining neurons within each visual area are ordered based on their partition similarity while the module IDs across different multi-resolution modular partition maps are matched using a heuristic algorithm based on their similarity for visual comparison. (C) The heatmap of pairwise adjusted rand index (ARI) between visual stimuli for session 7. Each multi-resolution modular partition map is considered a single clustering result, and ARI is used to measure the similarity between different partition maps. Note that ARI is independent of the color-matching heuristic algorithm and is thus more reliable. (D) The heatmaps of pairwise ARI between visual stimuli for two other mice (session 3 and session 4). Despite individual differences, the similarity between natural scenes and movies is always among the highest.

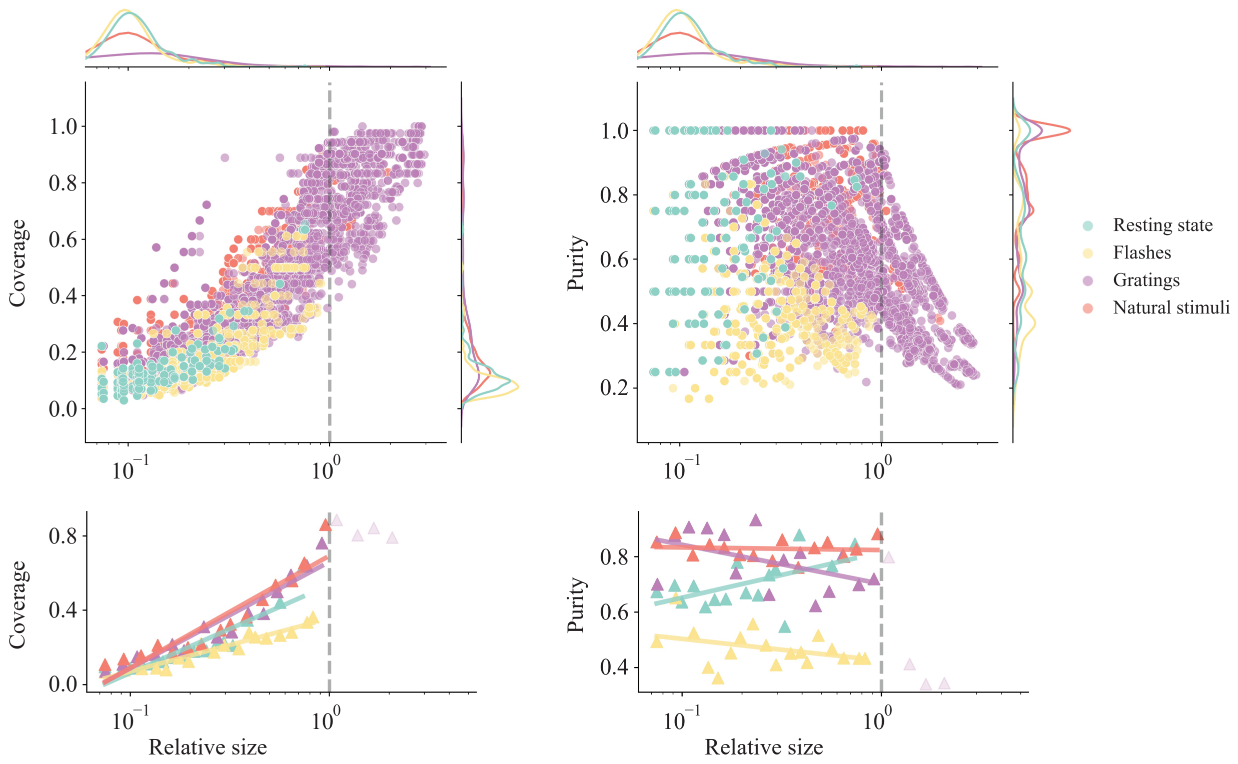


**Fig.** S15 | Fundamental properties of modular structure with module size. (top) Coverage/purity against relative module size for four stimulus groups where each dot represents a single module; relative module size is defined as module size divided by the largest area size. (bottom) Coverage/purity against relative module size with regression after log binning. Modules with a relative size larger than 1 are excluded in regression since their coverage/purity will introduce bias.

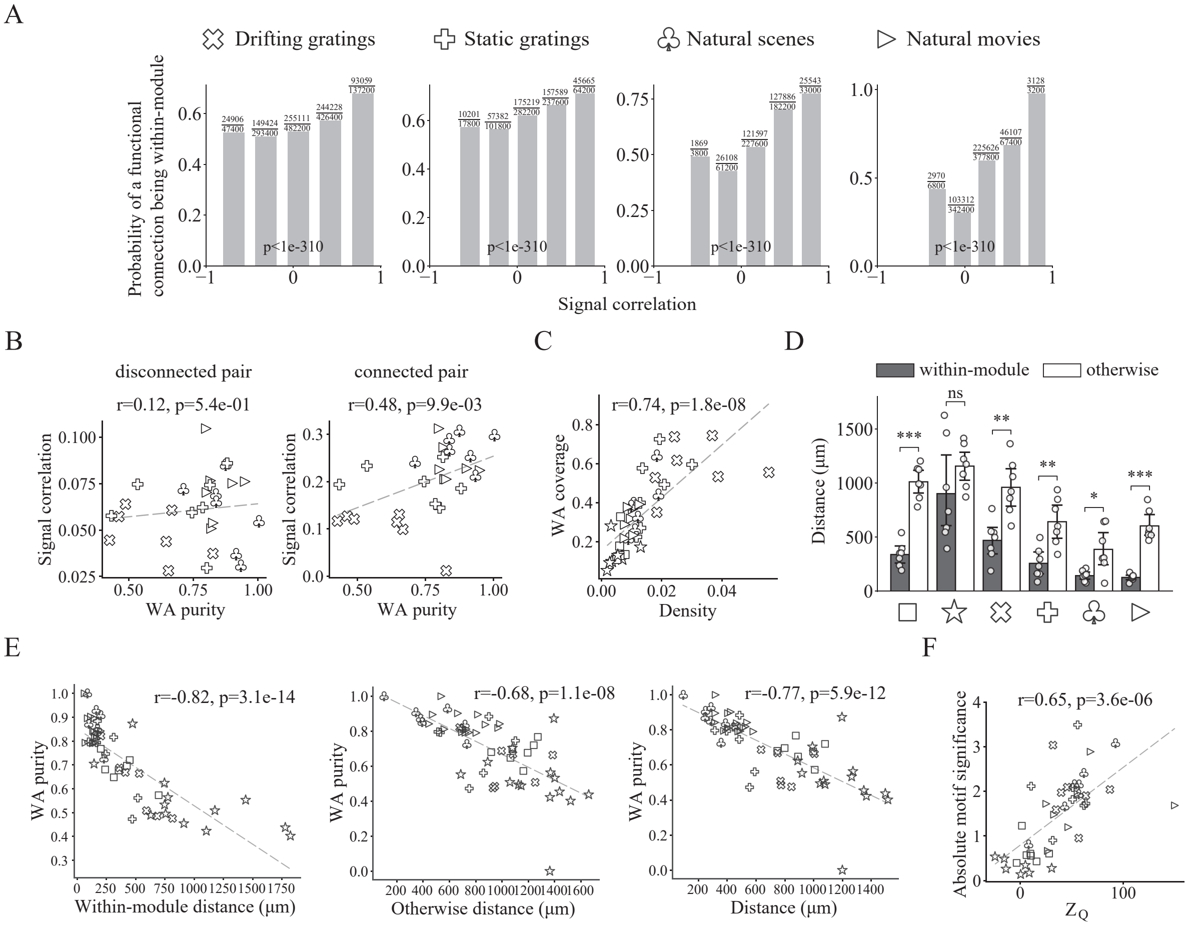


**Fig.** S16 | Biological interpretation of the modular structure. Two-sided Wald test is used in (B), (C), (E) and (F). (A) Probability of finding connected neuron pairs inside the same module against their signal correlation, Cochran-Armitage trend test, two-sided. (B) Linear regression results of signal correlation of chunked tuning curves with equal length against WA purity for (left) disconnected neuron pairs and (right) connected neuron pairs. Tuning curves are chunked into sequences with equal lengths for a fair comparison across stimuli, each dot represents the functional network of a mouse during certain stimulus presentations. (C) Linear regression results of WA coverage against network density. (D) Physical distance between connected neuron pairs that either belong to the same or not. ns $p > 0.05$, $*p < 0.05$, $**p < 0.01$, $***p < 0.001$, rank-sum test, one-sided, $n = 7$ mice. Error bars represent 95% confidence interval. (E) Linear regression results of WA purity against average distance between (left) within-module connected neuron pairs, (center) other connected neuron pairs and (right) all connected neuron pairs. Each dot represents the functional network of a mouse during certain stimulus presentations. (F) Average absolute motif significance (absolute Z-score of intensity) across all signed motifs against Z-score of Modularity.

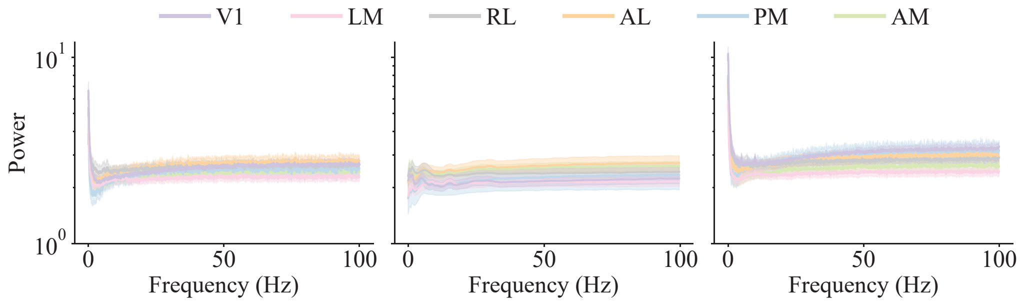


**Fig.** S17 | Average power spectrum of spike trains for each visual area from session 2 during various types of visual stimuli. Power spectrum is obtained through multi-taper Fast Fourier Transform. Due to the limited presentation duration of other stimuli (250 ms), only spike spectra during resting state, drifting gratings and natural movies are shown.

## References

[1] Allen Institute. Allen brain observatory – neuropixels visual coding, technical white paper: overview. *https://brainmapportal-live-4cc80a57cd6e400d854-f7fdcae.divio-media.net/filer_public/80/75/8075a100-ca64-429a-b39a-569121b612b2/neuropixels_visual_coding_-_white_paper_v10.pdf*, 2019.

[2] Guang Yong Zou. Toward using confidence intervals to compare correlations. *Psychological methods*, 12(4):399, 2007.

[3] Julie A Harris, Stefan Mihalas, Karla E Hirokawa, Jennifer D Whitesell, Hannah Choi, Amy Bernard, Phillip Bohn, Shiella Caldejon, Linzy Casal, Andrew Cho, et al. Hierarchical organization of cortical and thalamic connectivity. *Nature*, 575(7781):195–202, 2019.
