# Supplementary material for: Stimulus type shapes the topology of cellular functional networks in mouse visual cortex
Source: Nat Commun. 2024 Jul 9;15:5753. doi: 10.1038/s41467-024-49704-0 (PMC11233648; doi:10.1038/s41467-024-49704-0)
Supplement: Supplementary file 3 — Reporting Summary [file 41467_2024_49704_MOESM3_ESM.pdf]

nature portfolio

Corresponding author(s): Disheng Tang

Last updated by author(s): May 4, 2024

Reporting Summary

Nature Portfolio wishes to improve the reproducibility of the work that we publish. This form provides structure for consistency and transparency in reporting. For further information on Nature Portfolio policies, see our Editorial Policies and the Editorial Policy Checklist.

Statistics

For all statistical analyses, confirm that the following items are present in the figure legend, table legend, main text, or Methods section.

n/a	Confirmed	
☐	☒	The exact sample size (*n*) for each experimental group/condition, given as a discrete number and unit of measurement
☐	☒	A statement on whether measurements were taken from distinct samples or whether the same sample was measured repeatedly
☐	☒	The statistical test(s) used AND whether they are one- or two-sided<br>*Only common tests should be described solely by name; describe more complex techniques in the Methods section.*
☐	☒	A description of all covariates tested
☐	☒	A description of any assumptions or corrections, such as tests of normality and adjustment for multiple comparisons
☐	☒	A full description of the statistical parameters including central tendency (e.g. means) or other basic estimates (e.g. regression coefficient) AND variation (e.g. standard deviation) or associated estimates of uncertainty (e.g. confidence intervals)
☐	☒	For null hypothesis testing, the test statistic (e.g. *F*, *t*, *r*) with confidence intervals, effect sizes, degrees of freedom and *P* value noted<br>*Give P values as exact values whenever suitable.*
☒	☐	For Bayesian analysis, information on the choice of priors and Markov chain Monte Carlo settings
☒	☐	For hierarchical and complex designs, identification of the appropriate level for tests and full reporting of outcomes
☒	☐	Estimates of effect sizes (e.g. Cohen's *d*, Pearson's *r*), indicating how they were calculated

Our web collection on statistics for biologists contains articles on many of the points above.

Software and code

Policy information about availability of computer code

Data collection	All the data analyzed in this manuscript is part of the Allen Brain Observatory which can be accessed from this link: https://allensdk.readthedocs.io/en/latest/visual_coding_neuropixels.html
Data analysis	Code for analyses in the manuscript and generation of figures are available from the repository: https://github.com/HChoiLab/functional-network

For manuscripts utilizing custom algorithms or software that are central to the research but not yet described in published literature, software must be made available to editors and reviewers. We strongly encourage code deposition in a community repository (e.g. GitHub). See the Nature Portfolio guidelines for submitting code & software for further information.

Data

Policy information about availability of data

All manuscripts must include a data availability statement. This statement should provide the following information, where applicable:

- Accession codes, unique identifiers, or web links for publicly available datasets
- A description of any restrictions on data availability
- For clinical datasets or third party data, please ensure that the statement adheres to our policy

All the data analyzed in this manuscript is part of the Allen Brain Observatory introduced in Joshua H Siegle, Xiaoxuan Jia, Severine Durand, Sam Gale, Corbett Bennett, Nile Graddis, Greggory Heller, Tamina K Ramirez, Hannah Choi, Jennifer A Luviano, et al. Survey of spiking in the mouse visual system reveals functional

hierarchy. Nature, 592(7852):86-92, 2021. The raw data used to generate main text Figs. 1–5 is available for download in Neurodata Without Borders (NWB) format via the AllenSDK. Example Jupyter Notebooks for accessing the data can be found at https://allensdk.readthedocs.io/en/latest/visual_coding_neuropixels.html. \textcolor{blue}{Source data for figures [1-5] are provided as a Source Data file.

Research involving human participants, their data, or biological material

Policy information about studies with human participants or human data. See also policy information about sex, gender (identity/presentation), and sexual orientation and race, ethnicity and racism.

Reporting on sex and gender	N/A
Reporting on race, ethnicity, or other socially relevant groupings	N/A
Population characteristics	N/A
Recruitment	N/A
Ethics oversight	N/A

Note that full information on the approval of the study protocol must also be provided in the manuscript.

Field-specific reporting

Please select the one below that is the best fit for your research. If you are not sure, read the appropriate sections before making your selection.

☒ Life sciences ☐ Behavioural & social sciences ☐ Ecological, evolutionary & environmental sciences

For a reference copy of the document with all sections, see nature.com/documents/nr-reporting-summary-flat.pdf

Life sciences study design

All studies must disclose on these points even when the disclosure is negative.

Sample size	We used 7 sessions (animals). The sample size is not determined through any statistical methods, the 7 out of 32 sessions in Stimulus Set 1 (Brain Observatory 1.1) are selected based on the prerequisite that recordings of all six visual areas (V1, LM, RL, AL, PM, AM) are available.
Data exclusions	We excluded the remaining sessions due to the lack of recordings of at least one of the six visual areas.
Replication	Reproducibility of the functional networks and their fundamental properties is ensured with statistical controls. The exact module partition and motif significance sequence may not be reproducible due to the stochastic nature of Louvain method and random graph generation via reference models. Nonetheless, the main findings and conclusions are still replicable considering the large number of repeats in our computation to reduce bias. All attempts at replication were successful.
Randomization	We gathered data from all animals without any selection criteria. Each animal's CCG was compared to surrogate in correlated data obtained via a uttering procedure applied to that same animal's data, to identify significant connections. There were no experimental manipulations (e.g. pharmacological interventions) for which randomization of animals into control vs treatment groups would apply.
Blinding	Connectivity graphs for each animal were constructed with a procedure that was fully specified before any network properties were analyzed. Thus the graph construction was done in a blinded manner with respect to the final results. Further analyses of these graphs (after their construction) were performed in a non-blinded manner.

Reporting for specific materials, systems and methods

We require information from authors about some types of materials, experimental systems and methods used in many studies. Here, indicate whether each material, system or method listed is relevant to your study. If you are not sure if a list item applies to your research, read the appropriate section before selecting a response.

Materials & experimental systems

n/a	Involved in the study
☒	☐ Antibodies
☒	☐ Eukaryotic cell lines
☒	☐ Palaeontology and archaeology
☒	☐ Animals and other organisms
☒	☐ Clinical data
☒	☐ Dual use research of concern
☒	☐ Plants

Methods

n/a	Involved in the study
☒	☐ ChIP-seq
☒	☐ Flow cytometry
☒	☐ MRI-based neuroimaging

Plants

Seed stocks	N/A
Novel plant genotypes	N/A
Authentication	N/A
